# Oral Branched-Chain Amino Acids Supplementation in Athletes: A Systematic Review

**DOI:** 10.3390/nu14194002

**Published:** 2022-09-27

**Authors:** Diogo V. Martinho, Hadi Nobari, Ana Faria, Adam Field, Daniel Duarte, Hugo Sarmento

**Affiliations:** 1 University of Coimbra, Research Unit for Sport and Physical Activity, Faculty of Sport Sciences and Physical Education, 3040-248 Coimbra, Portugal; 2 Dietetics and Nutrition, Coimbra Health School, Polytechnic of Coimbra, 3046-854 Coimbra, Portugal; 3 Laboratory for Applied Health Research (LabinSaúde), 3046-854 Coimbra, Portugal; 4 Department of Physiology, Faculty of Sport Sciences, University of Extremadura, 10003 Cáceres, Spain; 5 Department of Physical Education and Special Motricity, Faculty of Physical Education and Mountain Sports, Transilvania University of Braşov, 500068 Braşov, Romania; 6 School of Human and Health Sciences, University of Huddersfield, Huddersfield HD1 3DH, UK; 7 N2i—Polytechnic Institute of Maia, 4475-690 Maia, Portugal; 8 CIDESD—Maia University, 4475-690 Maia, Portugal

**Keywords:** athletic performance, exercise, recovery, protein intake

## Abstract

Branched-chain amino acids (BCAAs) are oxidized in the muscle and result in stimulating anabolic signals—which in return may optimize performance, body composition and recovery. Meanwhile, among athletes, the evidence about BCAA supplementation is not clear. The aim of this study was to review the effects of BCAAs in athletic populations. The research was conducted in three databases: Web of Science (all databases), PubMed and Scopus. The inclusion criteria involved participants classified both as athletes and people who train regularly, and who were orally supplemented with BCAAs. The risk of bias was individually assessed for each study using the revised Cochrane risk of bias tool for randomized trials (RoB 2.0). From the 2298 records found, 24 studies met the inclusion criteria. Although BCAAs tended to activate anabolic signals, the benefits on performance and body composition were negligible. On the other hand, studies that included resistance participants showed that BCAAs attenuated muscle soreness after exercise, while in endurance sports the findings were inconsistent. The protocols of BCAA supplements differed considerably between studies. Moreover, most of the studies did not report the total protein intake across the day and, consequently, the benefits of BCAAs should be interpreted with caution.

## 1. Introduction

Leucine, isoleucine and valine are three essential branched-chain amino acids (BCAAs) not produced by the body and, thus, should be exogenously consumed [1,2]. Notably, BCAAs bypass metabolism in the liver and are oxidized in skeletal muscle [3,4]. Leucine, in particular, activates the mammalian target of rapamciyn-1 (mTOR), an anabolic signal that mediates muscle protein synthesis [2,5], which in turn is related to adaptations in strength and hypertrophy [6]. To this end, it is hypothesized that BCAAs are beneficial for performance, recovery and body composition [7]. 

A recent statement claims that acceptable levels of BCAA intake should be prioritized with exercise participation [8]. However, oral ingestion of BCAAs is highly questionable with reference to optimizing performance and protein synthesis [6,9,10]. Recent systematic reviews show that BCAA supplementation tended to attenuate muscle soreness and is an indicator of muscle damage [10,11]. The ergogenic effects of BCAAs have mainly been examined in adults and few studies included trained participants, which may differ substantially in performance, muscle damage and body composition. Additionally, the Australian Institute of Sport classified BCAAs in group C, which include supplements without scientific support among athletes or inconclusive studies. However, BCAAs are not recommended within supplementation programs [7]. The overview of the International Olympic Committee did not mention BCAAs in the different topics discussed—namely, supplements used to prevent or treat nutrient deficiencies, supplements used for energy provision, and supplements that improve sports performance [12]. Concerning the three BCAAs, only the effects of leucine in protein turnover were described, although the studies mentioned did not include trained participants [13,14].

Systematic reviews are needed to establish decisions about the general application of sport supplements [15]. The effects of BCAA supplementation were previously examined in non-sport participants or combining samples of athletes and active participants. Therefore, a systematic review about the impact of BCAAs among athletes is needed. Given the potential anabolic impact of BCAA supplementation, the aim of this study was to review the effects on performance, body composition, recovery, biochemical, hormonal response and/or molecular signaling in athletes.

## 2. Materials and Methods

The current review was conducted according to PRISMA (Preferred Reporting Items for Systematic Reviews and Meta-analyses) guidelines [16] and Cochrane instructions [17].

### 2.1. Eligibility Criteria

The manuscripts included in the present review met the following criteria: (1) Population—athletes; (2) Intervention—oral ingestion of BCAAs; (3) Outcomes—indicators of performance, body composition, recovery, biochemical, hormonal response or molecular signalling; (4) Comparator—control group or placebo, and; (5) Output—pre- and post-test changes. The exclusion criteria were: (1) Studies including participants without relevant training experience; (2) Articles classified as a letter to the editor or review, and; (3) BCAA supplementation by infusion or combined with other substances.

### 2.2. Information Source and Search Strategy

Electronic databases (Web of Science (all databases), PubMed and Scopus) were searched on 11 February 2022. The search terms were adapted from previous systematic reviews about BCAA supplementation and exercise [10,11,18]. A recent narrative review about BCAAs strength and hypotrophy was also considered to define the search terms (6). The following search strategy was used: (“branched-chain amino acid*” OR “BCAA*” OR “branched-chain amino acid* supplementation” OR “BCAA* supplementation” OR valine OR isoleucine OR leucine) AND (strength* OR “resistance training” OR endurance* OR aerobic “intermittent sport” OR high-intensity OR “muscle soreness” OR “delayed onset muscle soreness” OR DOMS OR anabolic OR “protein synthesis” OR jump OR run OR sprint) AND (athlete* OR sport* OR train*). No restrictions were applied to the year of publication and only papers written in English were consulted.

### 2.3. Selection Process

Reference managing software (EndNoteTMX9, Clarivate Analytics, Philadelphia, PA, USA) was used to export the manuscripts. After the initial screening, duplicates were automatically removed and manually checked. Two independent authors (DVM/HS) screened the title and abstracts. The remaining manuscripts were assessed to check whether the eligibility criteria were met. Where disagreements occurred, a third author (AF) was consulted and debate ensued until a consensus was achieved.

### 2.4. Data Collection Process and Data Items

Two authors (DVM/HS) carefully examined the eligible papers and extracted the relevant information from each study. Thereafter, the sample population, BCAA supplementation (frequency and quantity), exercise protocol, outcomes of interest, aims and main results were summarized on a template adapted from Cochrane Consumers and Communication Review Group [19]. The outcomes were categorized as follows: performance, body composition, muscle soreness, recovery and biochemical, hormonal or molecular indicators. Two studies [20,21] used a similar sample to examine the effects of BCAA supplementation after an intense exercise protocol on different outcomes. The mentioned papers tested different hypotheses and, consequently, were included in the review. 

### 2.5. Study Risk of Bias

The revised Cochrane risk of bias tool for randomized trials (RoB 2.0) was used to analyse the risk of bias of randomized studies included in the present review [22], as suggested elsewhere [23,24]. The tool is formed by five domains with different questions: (1) Bias arising from the randomization process; (2) Bias due to deviations from intended interventions; (3) Bias due to missing outcome data; (4) Bias in measurement of the outcome; (5) Bias in selection of the reported result. In the present review, the effect of starting and adhering to the interventions as specified in the trial protocol option was selected in the tool since the present study examined pre- and post-test results of specific outcomes, considering BCAA supplementation and exercise intervention. The questions were signalled as: yes, probably yes, probably no, no and no information. Additionally, domains were individually signed as: low risk of bias, some concerns, and high risk of bias. Text boxes associated with each domain were completed when possible. Two independent observers completed the revised Cochrane risk of bias tool for randomized trials (DVM/HS) and possible disagreements were solved by a third reviewer (AF).

## 3. Results

**Figure 1:**
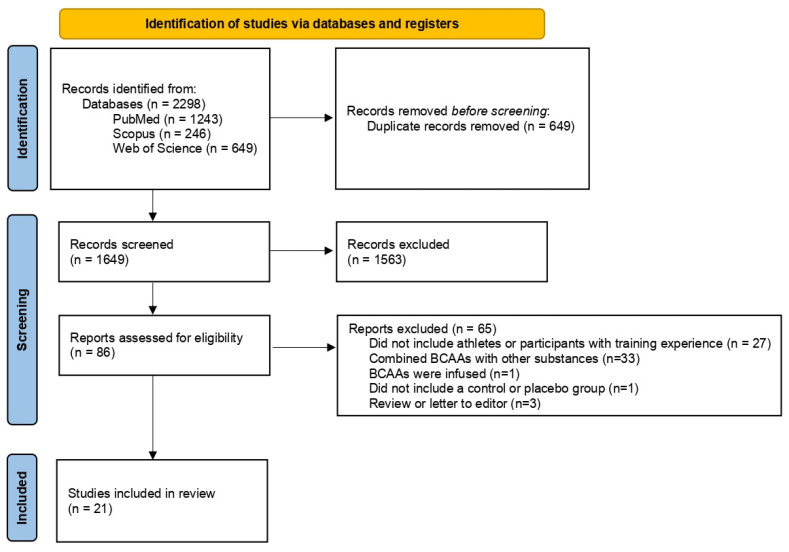
Flow chart diagram highlighting the studies included in the present review.

The electronic search identified 2298 records from the three databases. Following the removal of duplicates (n = 649), the remainder of titles and abstracts were screened. The full text of 86 papers were consulted and 65 were not considered in the present review for the following reasons: sample did not include athletes or participants with training experience (n = 27), BCAAs were combined with different substances (n = 33), one study administrated BCAAs by infusion, one study did not include a control group and three papers were classified as reviews or letters to the editor (Figure 1).

### 3.1. Study Characteristics

The studies included in the present review are summarized in Table 1. Different outcomes were used to examine the impact of BCAA supplementation among athletes. The studies have investigated the effect of BCAAs on performance [21,25,26,27,28,29,30,31], body composition [25,26], muscle soreness or recovery [21,25,26,27,28,29,30,31,32,33,34,35,36,37] and changes in biochemical, hormonal, or molecular signaling [20,21,25,26,27,28,29,30,33,34,36,38,39,40,41,42,43].

The effects of BCAAs were tested using different exercise protocols and/or exposing athletes to a variety of diets. Two studies examined the influence of BCAAs on specific outputs after an intense bout of exercise, promoting glycogenic depletion [20,21]. A muscle-damaging protocol was applied in resistance athletes and strength, jump performance, creatine kinase, muscle soreness were assessed after acute BCAA supplementation [36]. A separate investigation measured the impact of BCAAs on body weight, body composition, strength parameters and resting metabolic rate using a carbohydrate restrictive diet [27]. The BCAA supplementation protocols varied between studies in relation to timing and amount. For example, 10 g of BCAAs were ingested five minutes before and after strength training sessions [26], while immunological parameters were tested in 12 triathletes and 24 marathon runners across 1 month supplemented with six grams of BCAA [38].

### 3.2. Risk of Bias

**Figure 2:**
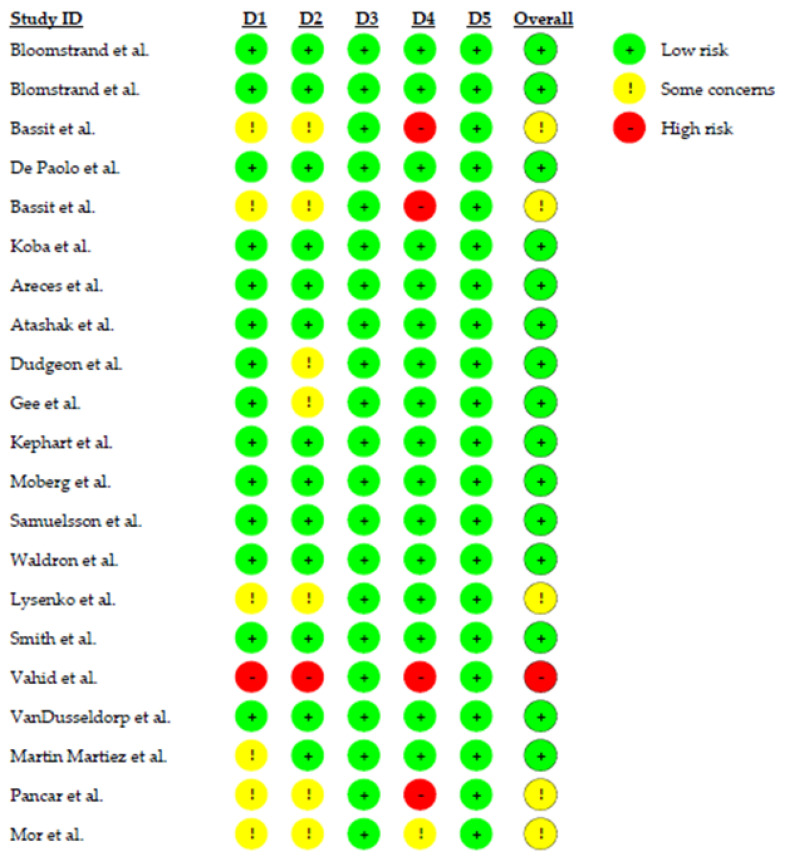
Risk of bias for each study [20,21,25,26,27,28,29,30,31,32,33,34,35,36,37,38,39,40,41,42,43] using the revised Cochrane risk of bias tool for randomized trials. D1: Bias arising from the randomization process, D2: Bias due to deviations from intended interventions, D3: Bias due to missing outcome data, D4: Bias in measurement of the outcome, D5: Bias in selection of reported results.

In general, studies show a lower risk of bias for the five domains (see Figure 2). The details of randomization were not totally clarified in studies [31,33,38,39,42]. The interpretation of the second domain—deviations from intended interventions—in seven studies [26,32,33,35,38,39,42] needs caution. On the fourth domain—concerning measurement outcome—five studies demonstrated a higher risk of bias [32,38,39,41]. This was mainly related to the lack of assessors blinding before the intervention or manuscripts failed to report whether observers were aware of treatment and control groups. The 21 studies show a low risk of bias in the third and fifth domains. 

### 3.3. Results of Individual Studies

For the studies that evaluated the impact of BCAA supplementation on performance, the participants typically consisted of cycling and running athletes [21,25,27,29], athletes undertaking resistance training [26,28,30], volleyball [31] and soccer players [32,33]. Allowing for variation in protocols of supplementation, the results of the present review suggest that BCAAs had trivial effects on performance outputs. In another study, participants followed a hypocaloric diet with either carbohydrate or BCAAs and a heavy resistance training programme across 8 weeks [26]. The authors found that BCAA supplementation increased upper (15.1 ± 2.2 kg) and lower body strength (7.1 ± 1.6 kg), significantly, while the CHO group demonstrated negligible changes in upper body strength (4.8 ± 1.8 kg). A separate study examining the effects of long-term BCAA supplementation over 10 weeks in 18 cyclists, found a group vs. time interaction for peak power in the BCAA group [27]. However, the authors did not control nutritional intake and athletes were instructed to maintain their habitual dietary habits during the investigation [27]. Other studies examined the effects of BCAA supplementation over 7 to 8 days [25,31,33] immediately before, during, or after exercise [21,28,29,30]. For these studies, few manuscripts tested BCAA supplementation with reference to performance indicators.

The effectiveness of BCAA supplementation on body composition was examined in 50 amateur runners [25], 17 resistance athletes [26] and 18 cyclists [27]. Comparable changes in body weight were observed in BCAAs and the control group after 7 days of oral supplementation [25]. Negligible effects for the BCAA group were observed in lean tissue and fat mass [26,27]. 

Muscle soreness or recovery were assessed by using scales or examining decrements in performance post-exercise through the use of specific field or laboratory tests. In endurance sports (running or cycling), the results of extracted studies are inconclusive. Ratings of perceived exertion and mental fatigue were significantly reduced in the BCAA group among seven endurance cyclists [21], while in 50 marathon runners no differences were reported in ratings of perceived exertion [25]. In contrast, muscle soreness was lower in the BCAA group compared with the placebo trial of 16 distance runners [34]. Studies that included resistance participants and examined the effects of BCAAs on muscle soreness or recovery indicated potential benefits of leucine, isoleucine or valine in the attenuation of muscle soreness after exercise [36,37]. Two studies reported that the consumption of BCAA supplementation attenuated the decrements in performance [36,37]. A variety of biochemical, hormonal, molecular, parameters were analyzed after an exercise intervention combined with BCAA supplementation. The main findings of these extracted studies reveal that BCAA intake caused a substantial improvement in BCAAs:tryptophan ratio [21,27] and immune response [27,38,39]. Allowing for methodological variation between studies, the hormonal changes indicate that BCAA supplementation favored an anabolic hormonal response. The cortisol level decreased after exercise and, in parallel, testosterone tended to rise in participants that engaged in resistance training [3]. Studies that examined biochemical mechanisms were mainly focused on specific metabolic signals and their dependence of target rapamycin complex 1 mechanism (mTORC1).

## 4. Discussion

The aim of this systematic review was to summarize the effects of BCAAs on performance, muscle soreness, recovery, body composition and biochemical, hormonal and molecular indicators among athletic populations. It was demonstrated that BCAA supplementation has minimal impact on performance and negligible effects on body composition. The effects of BCAAs on recovery and perceived muscle soreness were largely dependent of sport and exercise modality. For endurance sports athletes, the results differed between studies while in resistance participants the BCAAs mostly attenuated muscle soreness. 

An early study claimed that BCAAs can be used as fuel during exercise, as an alternative to carbohydrates and fats [43]. This may explain the adoption of BCAA supplementation during exercise in studies that evaluated performance outputs [21,28,29,30,32,33]. The effects of BCAAs on performance were negligible irrespective of short or long-term protocols of supplementation. Note, the skeletons of amino acids are often metabolized to Krebs cycle (citric acidy cycle) intermediates [44]. Although BCAA oxidation occurs mainly in the muscle, the impact of carbohydrates on the muscle is more evident. By inference, the role of carbohydrates on performance is crucial [45]. 

The contribution of BCAAs to promote protein synthesis and attenuate proteolysis is activated by the mTORC1, an anabolic signal, which is dependent of insulin and insulin-like growth factor 1 [2]. Among eight recreational active participants with at least one year of training experience, three weeks of BCAA supplementation combined with resistance training favored an anabolic profile with reference to cortisol and creatine kinase reductions, and an increased testosterone level [46]. This study excluded participants that exceeded 0.8 g.kg^−1^.day^−1^ of protein. More recently, contemporary research suggests that 1.6 g.kg^−1^.day^−1^ of protein intake is sufficient to optimize fat-free mass [47]. If an adequate intake of protein is ingested, the effects of BCAA on strength and hypertrophy are questioned [6]. The participants in the previous study [46], likely to meet the individual recommendations of protein intake and, consequently, the BCAA supplementation had a significant effect on cortisol, testosterone and creatine kinase. Comparable results were observed among 20 male soccer players 24-h post-strength session [48]. Although, insulin and testosterone were significantly higher in the BCAA group than the placebo, the daily protein intake per body mass was not reported [48]. In contrast, 30 resistance participants ingested approximately 1.67 g.kg^−1^ of protein and negligible differences were noted between the BCAA and placebo trials [30]. Another study [29] examined subsequent signals of mTORC1 activation—S6K1 (ribosomal protein kinase) and eukaryotic factor 4E binding protein—without reporting protein ingestion. However, studies that examine the effects of BCAAs should control the daily protein intake for intervention groups to ensure that robust inferences are obtained. 

When consuming during prolonged exercise, BCAAs showed an increase in the BCAAs:tryptophan ratio, confirming the central fatigue hypothesis [49]. It has been shown that during prolonged exercise, plasma fatty acids increased significantly and compete with tryptophan for binding to albumin. The increase in free tryptophan in the blood resulted in the transport of this amino acid across the blood–brain barrier. Consequently, tryptophan is converted into serotonin which is related to central fatigue. On the other hand, substantial increments of plasma BCAAs reduce the transportation of tryptophan to the brain and serotonin formation [45]. Two studies investigated the ratio BCAAs:tryptophan ratio during exercise [21,27]. For example, among 18 cyclists, the BCAAs:tryptophan ratio was higher in the BCAA group with an increase of 20% and 4% peak and mean power outputs, respectively [27]. It is plausible to suggest that the increase in BCAAs:tryptophan ratio reduced the production of serotonin and fatigue and, subsequently, performance improved. An additional study indicated differences in the BCAAs:tryptophan ratio between groups with a small impact on the Stroop Colour Word Test [21]. Nevertheless, BCAAs are often combined with other macronutrients or supplements and this issue needs future research.

The studies extracted from this systematic review suggest that BCAA supplementation attenuate muscle soreness, particularly in resistance athletes. Variable results were found as indicators of muscle damage (i.e., creatine kinase and lactate dehydrogenase) at post-exercise across studies. These results were consistent with previous reviews that examined the effects of BCAAs on muscle damage indicators and muscle soreness [10,18,50]. Muscle damage markers (creatine kinase, lactate dehydrogenase and myoglobin) were lower at 24 and 48 post-exercise for the BCAA group than the placebo condition. Likewise, delayed onset muscle soreness was lower for the BCAA group than the placebo trial [10]. A recent meta-analysis found that BCAAs had no significant effect on lactate dehydrogenase, while lower levels of creatine kinase were reported post-exercise in the BCAA group (<24 h; 24 h and 48 h). A significant heterogeneity between studies (75%) was observed 24- and 48-h post-exercise [10]. 

In the current review, the inclusion criteria did not include grey literature. Furthermore, the original studies present limitations that should be recognized. Firstly, few studies reported the nutritional intake, especially protein ingestion expressed per kilogram of body mass. Given the important role of leucine to simulate anabolic signals, future studies should analyze the effects of isolated leucine and BCAAs separately, by including different groups on experimental design. Additionally, future research should control the nutritional intake.

## 5. Conclusions

It was recently recommended that 200 mg.kg^−1^.day^−1^ over 10 days should be ingested to attenuate exercise-induced muscle damage [50]. In the present review, supplementation protocols were widely variable in terms of timing and quantity, and this issue deserves particular attention in future research. The present review highlighted that BCAA supplementation does not appear to have a significant impact on performance. On the other hand, oral ingestion of isolated BCAAs reduces muscle soreness. BCAAs are also available in different supplementation products (e.g., protein whey) and are often combined with other nutrients (e.g., carbohydrates). Thereby, the potential benefits of isolated BCAA supplementation among athletes to attenuate muscle soreness and delay fatigue need to be interpreted with caution. 

## Figures and Tables

**Table 1:** Characteristics of studies that included participants with training experience and branched-chain amino acid supplementation.

Study	Participants	BCAA Supplementation Protocol	Exercise Protocol	Objective	Main Results
Blomstrand et al. [20]	7 male endurance cyclists	Before and after every 15 min of exercise. Aqueous solution containing 7 g.L^−1^ (40% valine, 35% leucine and 25% isoleucine).	Cycling	Investigate the impact of BCAAs when muscle glycogen was reduced.	Biochemical, hormonal, molecular indicators: Plasma concentration of amino acids (during exercise): changes were noted for alanine and arginine after the ingestion of BCAAs; Muscle concentration of amino acids: valine, isoleucine and valine increased in the BCAA group; Decrement in glutamate when BCAAs were ingested; Muscle glycogen decreased significantly, during exercise, in the placebo trial.
Blomstrand et al. [21]	7 male endurance cyclists	Before and after every 15 min of exercise. Aqueous solution containing 7 g.L^−1^ (40% valine, 35% leucine and 25% isoleucine).	Cycling	Investigate the impact of BCAAs when muscle glycogen was reduced.	Performance: Stroop Colour Word Test—color task: The BCAA group performed better than the placebo trial; during word and color-word examinations, no differences were noted; No differences were noted between the trials in regards to the amount of work completed during the last minutes. Muscle soreness and recovery: During exercise, ratings of perceived exertion and mental fatigue were higher in the placebo trial than the BCAA group. Biochemical, hormonal, molecular indicators: No significant changes between BCAAs and placebos in oxygen uptake, respiratory exchange ratio, heart rate; Free tryptophan/BCAA ratios increased in the placebo during after exercise. The ratio remained unchanged in the BCAA group; Lactate increased in the BCAA trial; Muscle glycogen decreased significantly, during exercise, in the placebo trial.
Bassit et al. [39]	12 male triathletes	Thirty days before and one week after competition. After the training session (6.0 g: 60% leucine, 20% valine, and 20% isoleucine), during the first 30 days, and a single dose of 3.0 g 30 min before the triathlon, as well as a single dose (3.0 g) daily, in the morning, in the first week after the competition.	Running	Examine immunological parameters before and after an international competition.	Biochemical, hormonal, molecular, indicators: Glutamine decreased by 22.8% after competition in the placebo trial; Plasma glutamine remained unchanged in the BCAA group; Decrement in glutamine was associated with a higher rate of infection symptoms and a proliferative response of lymphocytes.
De Palo et al. [40]	11 male triathletes	Chronic treatment (one month)—0.2 g.kg^−1^ of body mass: leucine (1.17 g), isoleucine (0.63 g); valine (0.61 g) before each meal. Thirty minutes before exercise: oral dose—leucine (9.64 g), isoleucine (4.68 g), valine (0.61 g). before exercise: 30 min.	Running	Test the effect of chronic treatment with BCAA supplementation on lactate and growth hormones before and 60 min after running protocol.	Biochemical, hormonal, molecular indicators: After one month of BCAAs, lactate levels were lower than baseline; Growth hormone and growth hormone–binding protein also increased after one month of BCAA supplementation.
Bassit et al. [38]	12 male triathletes and 24 marathon runners	Thirty days before and one week after competition. BCAA was supplemented twice a day after each training session, to the triathletes or as a single dose to the runners (6.0 g of 60% leucine, 20% valine, and 20% isoleucine); single dose (3.0 g)—30 min before the competition; triathletes received 3.0 g of BCAAs one week after exercise.	Running	Examine immunological parameters before and after an international competition in triathletes and marathon runners.	Biochemical, hormonal, molecular indicators: Plasma glutamine decreased in the placebo trial; Plasma glutamine remained unchanged in the BCAA group and increased the proliferative response of blood mononuclear cells; BCAA supplementation stimulated the production of inter-leukin 2 and interferon. Additionally, it promoted a Th1 immune response.
Koba et al. [34]	16 male distance runners	Five days: 5 g leucine, 2.5 g isoleucine and 2.5 g valine per day.	Three timed training sessions (total 40 km) per day	Examine the effects of BCAAs on soreness and indicators of muscle damage.	Muscle soreness or recovery: Muscle soreness was lower in the BCAA trial. Biochemical, hormonal, molecular indicators: No differences in creatine kinase, lactate dehydrogenase and aldorase were noted.
Areces et al. [25]	50 marathon runners (n = 47 males; n = 3 females)	Seven days prior to the marathon race: 5 g.day^−1^ of BCAA (1:0.5:0.5 of leucine:isoleucine:valine) dissolved in 250 mL of water. Supplementation should be consumed in the hours following their habitual training routines.	Marathon race	Investigate the effectiveness of BCAA supplementation during 7 days to attenuate muscle damage.	Performance: No differences in running speed, jumping height, leg muscle, strength and power were noted between BCAAs and control group. Body composition: Similar changes in body weight in BCAAs and control trials. Muscle soreness or recovery: No differences in the rate of perceived exertion. Biochemical, hormonal, molecular indicators: Urinary myoglobin did not differ between groups.
Atashk et al. [41]	20 male soccer players	Thirty minutes before the exercise protocol: 200 mg.kg^−1^ of BCAA (50% leucine, 25% isoleucine, 25% valine).	Resistance exercises: high pull, lateral pull-down, standing overhead press, leg extension, leg curl, leg press, and bench press.	Investigate the supplementation of BCAA in acute hormonal responses.	Biochemical, hormonal, molecular, indicators: Immediately after and 1 h post-exercise testosterone was higher in the BCAA trial; Insulin concentration was higher 1 and 2 h post-exercise in the BCAA trial; No differences were noted in cortisol concentrations 1 and 2 h after exercise.
Dudgeon et al. [26]	17 male resistance training athletes	Eight weeks: 14 g of BCAA (prior and following each workout).	Four days per week of resistance training during 8 weeks.	Examine BCAA supplementation in body composition, strength during carbohydrate restriction.	Performance: Lower and upper body strength increased significantly in the BCAA group; Repetitions in fatigue (squat and bench press exercises) did not change in BCAA exposure. Body composition: Body mass and lean body mass did not change in the BCAA group; Fat mass decreased trivially in the BCAA trial.
Gee et al. [35]	11 male resistance training athletes	20 g of BCAA (2:1:1 leucine, isoleucine, valine). Five minutes before (10 g) and five minutes after strength training (10 g).	Multi-joint exercises: back squat, bench press, deadlift, military press, barbell row.	Test the contribution of BCAA on performance and muscle soreness after strength training.	Muscle soreness or recovery: Muscle soreness was comparable in the BCAA and placebo trials; BCAA intervention attenuated the decrement in jumping performance and seated shot-put throw.
Kephart et al. [27]	18 male cyclists	Ten weeks: 12 g of BCAA: leucine (6 g); isoleucine (2 g); valine (4 g).	160 km cycling per week.	Investigate a long-term intervention of BCAA in performance and immune system.	Performance BCAAs grouped increased peak power (20%) and mean power (4%) from pre to post-test. Mean power differences were not statistically different between groups; The BCAA group improved the time to complete 4 km by 11% but without statistical significance. Body composition: BCAAs did not have an impact on body fat percentage, fat mass and lean leg mass. Biochemical, hormonal, molecular indicators: The BCAA group had a greater post-BCAAs:tryptophan ratio compared to the placebo group; After intervention, neutrophils increased in the placebo intervention.
Moberg et al. [28]	8 male resistance training participants	BCAA supplement (110 mg.kg^−1^): 25% L-isoleucine, 45% L-leucine, and 30% L-valine. Leucine was given at a dose of 50 mg.kg^−1^. Drink with supplement (150 mL) before and after the warm-up sets, following the fourth and eighth sets and after 15, 30, 60, 90, and 120 min of recovery (total volume: 1.35 L).	Leg press (starting at 85% of their 1 RM; gradually reducing the load so that they could perform at least 8–12 repetitions to fatigue).	Test the BCAA intake in stimulation of mechanistic targets of rapamycin complex 1 (mTORC1)–anabolic signalling.	Performance: Participants performed a comparable number of repetitions in different groups (placebo, leucine, BCAAs and essential amino acids). Biochemical, hormonal, molecular indicators: The anabolic signal (70-kDA ribosomal protein S6 kinase) was greater in the BCAA trial than in the placebo group; Essential amino acids promoted greater anabolic signs (phosphorylation of eukaryotic translation initiation factor 4E binding protein 1) than BCAAs; During recovery, differences between BCAAs and essential amino-acids were minimal.
Samuelsson et al. [29]	8 male resistance training participants	BCAA supplement (110 mg.kg^−1^): 25% L-isoleucine, 45% L-leucine, and 30% L-valine. Administered 150 mL before and after the warm-up sets, following the fourth and eighth sets and after 15, 30, 60, 90, and 120 min of recovery (total volume: 1.35 L).	The subjects performed 10 sets × 8–10 repetitions with rest between sets (3 min).	Test the BCAA intake in stimulation of PGC-1α4 (a potential regulator of muscle hypertrophy).	Performance No differences were noted in total work performed between trials. Biochemical, hormonal, molecular indicators: P70S6 kinease phosphorylation (signal of mTORC1) was greater in BCAAs and essential amino acids groups than placebo or leucine trials; PGC-1α4 (regulator of muscle hypertrophy) stimulation was reduced when BCAAs or essential amino acids were ingested.
Waldron et al. [36]	16 resistance training athletes (n = 14 males; n = 2 females)	Thirty minutes before and after the muscle damage protocol re-testing.	Seventy per cent of 1 RM for 10 repetitions across 6 sets.	Examine the effects of BCAA supplementation on recovery after an induced muscle damage protocol.	Muscle soreness or recovery: Decrement in muscle strength was attenuated 24 and 48 h post-exercise; Small differences (BCAAs > placebo) in counter-movement jump 24 and 48 h post-exercise; Muscle soreness was higher in the placebo trial 24 and 48 h post-exercise. Biochemical, hormonal, molecular indicators: Creatine kinase was higher 24 and 48 h post-exercise in the BCAA group.
Lysenko et al. [42]	9 endurance athletes	0.1 g.kg^−1^ immediately after exercise. BCAA in the form of capsules leucine, isoleucine, and valine: 2:1:1.	Cycling	Modulate post exercise anabolic and proteolytic signalling.	Biochemical, hormonal, molecular indicators: BCAAs surpassed the exercise inhibitor of mTORC; BCAAs surpassed a mitochondrial biogenesis signal: PG1-1α.
Smith et al. [30]	30 male resistance athletes	7.5 g of leucine, isoleucine, and valine. Before and after warm-up, and immediately following the last set of each exercise.	Barbell bench press, landmine bent-over row, barbell incline press, and landmine close-grip row (5 sets × until failure at 65% of 1 RM - 2 min of rest).	Examine the effects of CHO and/or BCAA supplementation during resistance training in hormonal responses and exercise performance.	Performance: No differences were noted between interventions in exercise repetitions. Biochemical, hormonal, molecular indicators: Cortisol levels were lower in the BCAA group than BCAAs combined with carbohydrates and placebo; Insulin concentration was significantly higher in the carbohydrate group than the BCAA and placebo group.
Vahid et al., [41]	30 track and field athletes	Forty-two days of supplementation.	Six weeks of athletics training.	Examine the effects of BCAA supplementation on fatigue.	Biochemical, hormonal, molecular indicators: BCAAs did not have an impact in two indicators of fatigue: lactate and ammonia.
VanDusseldorp et al. [37]	20 male resistance athletes	Eight days: 0.22 g.kg^−1^.day^−1^ during 8 days	Squat: 10 sets × 8 repetitions at 70% 1 RM.	Examine the effects of BCAA supplementation on recovery and muscle damage.	Muscle soreness or recovery: No differences were noted between the BCAA and placebo trials in vertical jump, jump squat after eccentric exercise; Maximal voluntary isometric contraction returned to baseline levels after exercise; The BCAA group reported less muscle soreness at 48 h and 72 h post-exercise. Biochemical, hormonal, molecular indicators: Creatine kinase was lower in the BCAA trial, 48 h after exercise, than the placebo group.
Martín-Martíez et al. [32]	12 male volleyball players	Five hundred milliliters of water containing a 2:1:1 ratio for leucine, isoleucine and valine (7 g). Three times during one week (Monday, Wednesday, Friday).	Volleyball training sessions: warm-up, plyometric, technical and tactical drills, cool-down.	Test the effect of BCAA supplementation on jump performance.	Performance: No statistical differences in counter-movement jump height between placebo and BCAAs groups were noted.
Pancar et al. [32]	14 male soccer players	Two grams before and immediately before exercise.	Five sets × 20 drop jumps.	Test BCAA supplementation on performance and recovery	Performance: No statistical differences in vertical jump between the placebo and BCAA groups were noted; No statistical differences in speed between the placebo and BCAAs groups were noted. Muscle soreness or recovery: No statistical differences in muscle soreness between the placebo and BCAA group were noted.
Mor et al. [33]	24 male soccer players	Seven days: 5000 mg BCAA (2500 mg 30–40 min before training and 2500 mg 1 h after training).	Soccer training	Examine BCAA and creatine supplementation on anaerobic capacity and ball shooting.	Performance: After intervention, mean power in the BCAA trial increased. Peak power and fatigue index decreased in the placebo group (no statistical differences were noted between groups).

## Data Availability

The data presented in this study are available in the present article.
